# Smoking Cessation Support for Pregnant Women Provided by English Stop Smoking Services and National Health Service Trusts: A Survey

**DOI:** 10.3390/ijerph19031634

**Published:** 2022-01-31

**Authors:** Ross Thomson, Sue Cooper, John Waldron, Efe Mamuzo, Lisa McDaid, Joanne Emery, Lucy Phillips, Felix Naughton, Tim Coleman

**Affiliations:** 1 Centre for Academic Primary Care, School of Medicine, University Park, University of Nottingham, Nottingham NG7 2RD, UK; sue.cooper@nottingham.ac.uk (S.C.); Lucy.Phillips1@nottingham.ac.uk (L.P.); tim.coleman@nottingham.ac.uk (T.C.); 2 Action on Smoking and Health, Unit 2.9, The Foundry, 17 Oval Way, London SE11 5RR, UK; john.waldron@ash.org.uk (J.W.); efe.mamuzo@ash.org.uk (E.M.); 3 School of Health Sciences, University of East Anglia, Norwich NR4 7UL, UK; L.Mcdaid@uea.ac.uk (L.M.); Joanne.Emery@uea.ac.uk (J.E.); f.naughton@uea.ac.uk (F.N.)

**Keywords:** smoking cessation, smoking in pregnancy, service evaluation, nicotine replacement therapy, online survey

## Abstract

Reducing smoking rates in pregnancy continues to be a public health priority. Given a recent UK government policy change resulting in The National Health Service (NHS) making a significant new contribution to cessation support in pregnancy in England, in addition to that of Local Authorities (LA), an up-to-date assessment of national support offered to pregnant women is needed. LA Stop Smoking Service (SSS) managers and representatives from maternity services in NHS Trusts were invited to participate in an online survey in autumn 2020. Topics included service configuration, staffing, practitioner consultations and treatments offered. The survey response rate was 66% (99/151) of LAs and 68% (95/140) of Trusts. Most LAs provided smoking support for pregnant smokers (78%), whereas under half (43%) of NHS Trusts did. Combination nicotine replacement therapy, i.e., a combination of a patch and short-acting product, was offered by LAs (92%) and Trusts (95%) and most commonly for 12 weeks duration, at 53% and 50%, respectively. Similar national online training was undertaken by those supporting women, with the majority undertaking the specialist pregnancy-specific module: LAs 60% and Trusts 79%. However, clinicians were reported to deliver specialist stop smoking support in over 50% of Trusts, whereas this was reported in only 16% of LAs. In England, both LA and NHS Trusts are currently delivering similar stop smoking support to pregnant women. Having nationally recognised treatment programmes and training allows for the delivery of consistent, evidence-based smoking cessation to pregnant women in different healthcare settings.

## 1. Introduction

Smoking during pregnancy is a leading yet preventable cause of adverse prenatal and birth outcomes, which also has detrimental health implications during childhood [1,2,3,4]. In England, around 10% of pregnant women are recorded as smoking at the time of delivery, but rates are from two to three times higher than this in younger women, those from the most deprived backgrounds and those in routine and manual occupations [5,6]. Before 2015, rates of smoking in pregnancy were falling, but since then, this decline has stalled [6,7]. Although the latest figures show a noticeable decrease in the percentage of mothers who were smokers at the time of delivery from the previous year, issues with the quality of the reported statistics indicate these data should be interpreted with care over the COVID-19 period and are still above the national ambition of 6% or less [8]. There are also differences in smoking at the time of delivery rates across English Clinical Commissioning Groups, with the lowest proportions reported in Central London (1.3%), West London (1.8%) and Hammersmith and Fulham (2.2%) and the highest reported in Blackpool (23.7%), North East Lincolnshire (22.8%) and Hull (20.5%) [5]. This highlights that efforts to reduce smoking rates in pregnancy should continue to be a public health priority, and access to effective interventions and specialist services is important [9].

Provision of stop smoking services for pregnant women began in 2000 in the UK, and the first guidelines on how to stop smoking in pregnancy were published in 2010 [10,11]. These guidelines recommended the use of psychosocial interventions and nicotine replacement therapy (NRT), which provides nicotine in a form that does not include the harmful elements found in tobacco smoke [11]. In 2013, the responsibility for public health and the commissioning of stop smoking services (SSSs) in England transferred from the National Health Service (NHS) to local government, and this has been accompanied by a decrease in NRT prescribing to pregnant women by General Practitioners [12]. In 2018, most local authorities (LA) commissioned a specialist SSS in which stop smoking advisers offer evidence-based behavioural support alongside pharmacotherapies to help people stop smoking as part of a programme of one-to-one or group support [13]. However, some offered integrated lifestyle/wellbeing services, which target multiple health behaviours such as diet, exercise and smoking together. Other LAs only commissioned stop smoking support from primary care General Practitioners and pharmacists, and a minority of local authorities decommissioned their services for smokers altogether [13]. A similar range of services for smokers was commissioned or provided by local authorities in 2020 [14].

The NHS Long Term Plan is a major new policy initiative, which, amongst other things, has committed the NHS to provide stop smoking support to pregnant women via a new smoke-free pregnancy pathway, which includes focused sessions and treatments [15], but how different English NHS Trusts are implementing this pathway is unclear. Given the changing face of Local Authority stop smoking service provision and that government policy is advocating for the implementation of stop smoking support in pregnancy to be provided through the NHS, an up-to-date assessment of the national support offered to pregnant women is needed.

To understand the current landscape of how English SSSs support pregnant women and investigate how the new NHS Long Term Plan is being implemented, we conducted two national surveys. We aimed to assess how SSS are configured within LAs and NHS Trusts and how they deliver smoking cessation support to pregnant women in England.

## 2. Materials and Methods

This work was conducted according to the tenets of the Declaration of Helsinki. As with previous studies [10,16], this survey was conducted as part of a service evaluation; therefore, ethical review was not required.

We designed two similar online surveys (see Figures S1 and S2) to reflect the differences in services; the first was intended to collect data from LA stop smoking services and the second from NHS Maternity Trusts. The design and content of both surveys were tested and piloted with two stop smoking practitioners, a smoking cessation service manager and a Maternity Stop Smoking Adviser, which only resulted in minor alterations. Topics included:

(a) *Service configuration*: We asked each organisation if they commissioned/provided specialist stop smoking support specifically for pregnant women. We asked what type of service is provided (e.g., specialist or integrated support), where the support is delivered and whether there is any joint funding of services.

(b) *Staffing and practitioner consultations*: We asked what kind of health professional was delivering the support, what training they had received to deliver the support and where initial and follow-up support appointments were delivered.

(c) *NRT and smoking cessation treatments*: We asked whether or not services had a budget for NRT, how this is supplied to pregnant women, what types are offered, how long for, and what advice is given on NRT use during a lapse to smoking. We also asked whether incentives are offered for successful quits and if Services offered e-cigarettes as a cessation support option.

The local authority survey was designed to be completed by the person responsible for day-to-day management of the smoking cessation service in each LA. The survey was distributed to the 151 local authorities in England as a module within a seventh annual survey of tobacco control leads in English local authorities commissioned by Cancer Research UK and conducted by Action on Smoking and Health (ASH). The survey was available for completion online during August and September 2020. Non-respondents were subsequently followed up by email reminders every two weeks until the survey closed.

The NHS survey was also distributed by ASH. At the time of this survey, there was no definitive list of NHS Trusts that provided stop smoking support to pregnant women, so an email invitation containing a link to the online survey was sent to a list of contacts who were either members of Action on Smoking and Health’s Smokefree Pregnancy Information Network or had attended events organised by ASH relevant to smoking in pregnancy. This resulted in invitations being sent to 140 English NHS Trusts that provided maternity care. The NHS survey was open for completion by individuals who oversaw the delivery of specialist smoking cessation support to pregnant women within their local NHS Trust between September and December 2020. If contacts felt they were not the appropriate person to complete the survey, they were encouraged to forward the invitation email to more suitable colleagues and inform ASH. Non-respondents were contacted every two weeks with reminder emails until the survey closed.

All survey responses were collected over encrypted connections, and respondents were informed that their responses would remain confidential and non-identifiable. Due to the General Data Protection Regulation Law, we were unable to access information on the characteristics of non-respondents that did not leave contact details. All questions referred to the commissioning/financial period between April 2020 and March 2021. As not all survey questions were mandatory, not all respondents completed all questions; therefore, there are some missing responses. In addition, some question types allowed for multiple responses.

Statistical analyses were conducted using SPSS version 27 [17]. We present descriptive analyses to summarise the interventions and support provided to women.

## 3. Results

For the LA survey, 99 complete responses were received from the 151 English local authorities that were invited to take part (66%). Most respondents identified their job roles as either Tobacco Control Lead (26%), Commissioner of tobacco control/smoking cessation services (19%) or holding both roles (38%). For the NHS survey, ASH sent surveys to contacts in maternity services located in 140 English NHS Trusts, and responses were received from 95 Trusts (68%). Of those that completed the survey, most identified their job roles as Smoking Cessation Specialist Midwives (37%) or Smoke Free team leaders (37%). The regions covered by Local Authorities and NHS Trusts that completed surveys are presented in Table 1.

### 3.1. Service Configuration

Most local authorities provided intensive smoking support, including carbon monoxide monitoring, behavioural support and pharmacological interventions for pregnant smokers (78%), whereas under half (43%) of NHS Trusts did. Seventy-nine per cent of LAs reported providing a specialist stop smoking service. Of the 41 NHS respondents who indicated that their Trusts did currently commission intensive stop smoking support for pregnant smokers, 11 were not responsible for overseeing the day-to-day delivery of intensive smoking cessation support to pregnant women and were therefore unable to complete the rest of the survey. Of those NHS Trusts that provided specialist stop smoking support, 67% reported that this was jointly funded with local authorities and that 57% provided support in hospitals.

### 3.2. Staffing and Practitioner Consultations

Fifty-one per cent of local authority stop smoking services reported employing practitioners who worked only or predominately with pregnant women to provide the majority of cessation support, with a similar amount (47%) working with all smokers. In NHS Trusts, 56.6% reported using smoking cessation practitioners with no other clinical responsibilities to deliver stop smoking support, while 90% of Trusts reported that midwives or healthcare assistants deliver this support (see Table 2).

The smoking cessation training of staff delivering support to pregnant women and how they deliver that support is shown in Table 3. Overall, the training of practitioners who support pregnant women to stop smoking is similar in Local Authorities and NHS settings. The use of online pregnancy-specific training, both brief advice and full training, is reported as 83% and 80%, respectively, by Trusts and 61% and 60% by LAs. Support was delivered in similar ways in both LA and NHS settings. One-to-one support delivered remotely, by either telephone or video call, was the most common type of support offered by both LAs (80%) and NHS Trusts (83%) for initial consultations. This was also true for follow-up appointments by LAs (82%) and Trusts (87%).

### 3.3. NRT and Smoking Cessation Aids

Table 4 shows how Nicotine Replacement Therapy and other smoking cessation treatments are utilised by NHS Trusts and LAs. A third of Trusts that offered stop smoking support to pregnant women (33%) reported not having a budget for NRT, as did 11% of LAs that also offered support. Most services that reported providing NRT to pregnant women offered them combination therapy (i.e., a long-lasting patch along with a short-acting NRT product): LAs (92%) and Trusts (95%). Only one trust reported offering monotherapy in the form of a short-acting product only. NRT was most commonly offered for 12 weeks by LAs (53%) and Trusts (50%), although around a quarter of LAs and Trusts continued to offer NRT in excess of 12 weeks. LAs (32%) and Trusts (60%) expected practitioners to advise pregnant women to continue using NRT during a brief lapse. Some LAs and Trusts provided e-cigarettes to pregnant women to assist them with cessation (11% and 7%, respectively); using incentives to promote successful quit attempts was also unusual (14% and 17%, respectively).

## 4. Discussion

These surveys give a contemporary picture of how Local Authorities and NHS maternity services’ commissioned stop smoking services for pregnant women in England offer support, providing insight into the similarities and differences in approach the different services take. Despite major differences between organisations, recently developed NHS stop smoking support is very similar to the more established support offered by LAs. NHS Trusts’ stop smoking support appears more likely to be delivered by clinicians, but direct provision of combination NRT is the pharmacological treatment of choice across both sets of services, and both make limited use of e-cigarettes or financial incentives to aid smoking cessation.

A 70% response rate is reasonable in a study of this kind [18], and even though we received responses from the majority of the organisations surveyed, we cannot comment on what is happening in LAs or NHS trusts for which there was no response. We also cannot guarantee that the surveys were completed by people with oversight of the support provided; however, we think it unlikely that people who were not familiar with support would have taken time to respond. While we could not find a definitive list of NHS Trusts that deliver ante-natal care, we received 95 responses, which is 71% of the 133 Trusts that submit data to the Hospital Episodes Statistics that are used to compile the NHS Maternity Statistics reporting on maternity activity in English NHS hospitals [19]. This gives us some confidence that the results are representative. Although we acknowledge that because the list of Trusts used was compiled from those with a specific interest in smoking cessation, we were unable to identify the number of NHS maternity services that offer no support and so could have resulted in an overestimation of the proportion of all NHS trusts that provided stop smoking support. This will not, however, have influenced descriptions of the kinds of support provided. As with all surveys, some questions may have been misinterpreted, and while we attempted to simplify the terminology and provided additional information for questions where appropriate (for example, a definition of single and combination NRT), we cannot be sure all items were similarly understood. We hope to have mitigated these potential shortcomings by addressing any issues that were raised during the piloting of the surveys. Responses relating to remote delivery of support should be viewed with caution given the timing of the survey with COVID-19 restrictions and adapted ways of working. While we did not design these surveys to make statistical comparisons between services or regions, a particular strength of this study is that, to our knowledge, this is the first survey since the Long Term Plan directed the English NHS to provide specialist smoking cessation support to pregnant women, so findings are novel.

In line with previous surveys of English NHS Primary Care Trusts in 2011 [10] and LAs in 2015 [15] that focused on stop smoking support provided specifically to pregnant women, we also found that, while we did not apply formal statistical tests, NRT was the most frequently offered treatment (92%). However, since 2011, there appears to have been an increase in services issuing pregnant women with NRT products directly. Around half of LAs and Trusts reported supplying NRT directly to pregnant women. While there is no evidence that how NRT is supplied affects long-term quitting rates in pregnancy, outside of pregnancy, ease of access to nicotine products can increase the use of NRT and significantly impact short-term quitting rates [20] and so may help women on their quitting journey.

In 2015 [16], there was considerable uncertainty amongst SSS managers about whether to advise pregnant women to use e-cigarettes, with only 8% of services likely to advise pregnant women to either start or continue using e-cigarettes. In our current survey, 11% of LAs are able to provide e-cigarettes as a cessation aid. While there is evidence that e-cigarettes may be useful in helping people quit smoking in the general population [21], there are mixed findings for vaping in relation to smoking cessation in pregnancy [22]. There is, however, the potential for vaping products to reduce the negative health outcomes associated with smoking, and the current guidance in the UK is that vaping should be supported if it helps pregnant women avoid smoking [23].

Findings highlight the considerable similarities between how NHS Trusts and LAs are delivering support; a potential explanation for this is that, in England, the National Centre for Smoking Cessation and Training (NCSCT) issues guidance related to the quality of stop smoking support. In 2019, the NCSCT published the ‘Standard Treatment Programme for Pregnant Women’, which describes the components of evidence-based, structured smoking cessation interventions intended for use with pregnant women treated within the English health system [24]. The NCSCT also provide practitioners’ skills-based training modules in delivering stop smoking support. The similarity between LA and NHS Trusts’ support methods and the utilisation of the same skills-based training packages illustrate the value of having a national centre to set standards of training and define treatments, all of which promote better clinical practice and better cessation outcomes for smokers [25] as well ensure the consistent factual messaging from health care professionals, valued by pregnant women [26].

One major difference between the support offered by LAs and NHS Trusts is that clinicians (midwives, nurses or health care assistants) were reported to deliver specialist stop smoking support in over 50% of Trusts, whereas specialist support was delivered by clinicians (GPs and pharmacists) in only 16% of LAs. Previous work has shown that, while pregnant smokers value receiving stop-smoking advice from midwives and tend to have a negative view of stop smoking services [27], midwives hold mixed views on delivering stop smoking support and talk of tension between maintaining a positive relationship with the woman and addressing the issue of smoking [28]. However, Midwives are especially well placed to deliver stop smoking support to pregnant women, and there is growing evidence that stop smoking interventions provided by specially trained midwives and maternity staff at antenatal appointments has the potential to increase smoking cessation [29].

Given that this is the first time NHS Trusts’ specialist stop smoking support for pregnant women has been surveyed since the recent rollout of the NHS Long-Term Plan, we believe that similar surveys repeated regularly over a number of years will provide a snapshot of how support develops over time based on national standards, which can be linked to outcomes and ultimately identify innovative and best practice.

## 5. Conclusions

This work illustrates that, in England, both Local Authority and NHS Trusts are currently delivering similar stop smoking support to pregnant women. With a clearly defined standard regarding what smoking cessation support should be provided to pregnant women and a nationally recognised training programme for any professional involved in delivering that support, it is possible for health providers to ensure that the support commissioned by varied organisations is both consistent and evidence-based. The continued rollout of support within NHS Trusts merits regular monitoring of service delivery.

## Figures and Tables

**Table 1:** Regions covered by Local Authorities and NHS Trusts.

Regions Covered	LA Services *n* (%)	NHS Trusts Services *n* (%)
Number of Respondents	*n* = 99 LAs	*n* = 30 NHS Trusts
East of England	7 (7%)	3 (10%)
London	20 (20%)	2 (6%)
East and West Midlands	14 (14%)	5 (17)
North East, Yorkshire and Humberside	24 (24%)	8 (27%)
North West	9 (9%)	5 (17%)
South East	14 (14%)	5 (17%)
South West	11 (11%)	2 (6%)

**Table 2:** Staff providing support to pregnant women.

Staff Providing Smoking Cessation Support for the Majority of Pregnant Women, *n* (%)	
**LAs**	***n*** = **99**
Stop smoking specialist(s) working only or mainly with pregnant women	51 (51%)
Stop smoking specialist(s) working with all smokers, including pregnant women	47 (47%)
GPs or Pharmacists	16 (16%)
Lifestyle/wellbeing counsellor(s)	7 (7%)
Other	27 (27%)
**NHS Trusts**	***n*** = **30**
Stop smoking practitioners who are not otherwise clinically trained	17 (57%)
Specially trained midwife/nurse	16 (53%)
Specially trained healthcare assistants	11 (37%)

**Table 3:** Overview of staff training and support delivery.

Staffing and Support Type	LA Services *n* (%)	NHS Trusts Services *n* (%)
Number of Respondents	*n* = 99 LAs	*n* = 30 NHS Trusts
What smoking cessation training have Practitioners who see pregnant women undergone? (tick all that apply)		
*‘Very Brief Advice on Smoking’—online*	60 (60%)	23 (79%)
*‘Very Brief Advice on Smoking for Pregnant Women’—online*	61 (61%)	24 (83%)
*‘Stop Smoking Practitioner Training’—online*	76 (76%)	23 (79%)
*‘Pregnancy and Smoking Cessation’—online*	60 (60%)	23 (79%)
*Face-to-face training delivered by NCSCT trained staff*	41 (41%)	17 (65%)
*Don’t know*	12 (12%)	4 (15%)
*Other*	24 (24%)	9 (35%)
How are initial stop smoking support appointments with pregnant women conducted? (tick all that apply)		
*Individual face-to-face*	70 (70%)	18 (60%)
*Group face-to-face*	2 (2%)	0 (0%)
*Remote (telephone/video call)*	80 (80%)	25 (83%)
*Other*	30 (30%)	6 (20%)
How are follow-up stop smoking support appointments with pregnant women conducted? (tick all that apply)		
*Individual face-to-face*	73 (73%)	21 (70%)
*Group face-to-face*	3 (3%)	1 (3%)
*Remote (telephone/video call)*	82 (82%)	26 (87%)
*Other*	28 (28%)	5 (17%)

**Table 4:** NRT and smoking cessation aids.

NRT and Smoking Cessation Aids	LA Services *n* (%)	NHS Trusts Services *n* (%)
Number of Respondents	*n* = 99 LAs	*n* = 30 NHS Trusts
Does your service have a budget for or provide NRT to pregnant women?		
*Yes*	84 (84%)	20 (67%)
*No*	11 (11%)	10 (33%)
*Missing*	5 (5%)	*N*/A
How is NRT supplied to pregnant women? (tick all that apply)	***n*** = **84**	***n*** = **20**
*Direct provision of NRT from your service*	43 (51%)	11 (55%)
*Voucher for NRT to be redeemed at a local pharmacy*	39 (46%)	9 (45%)
*GP prescription*	18 (21%)	*N*/A
*Hospital prescription (i.e., redeemed at hospital pharmacy)*	*N*/A	1 (5%)
*Other*	9 (10%)	3 (15%)
What types of NRT do you offer pregnant women?	***n*** = **84**	***n*** = **20**
*Combination NRT*	77 (92%)	19 (95%)
*Fast-acting only*	0	1 (5%)
*Patch only*	0	0
*Missing*	7 (8%)	*N*/A
*Number of weeks NRT is offered to pregnant women*	***n*** = **84**	***n*** = **20**
*Less than 12 weeks*	10 (12%)	5 (25%)
*12 weeks*	45 (53%)	10 (50%)
*More than 12 weeks*	20 (24%)	5 (25%)
*Not known/missing*	24 (28%)	*N*/A
Does your service offer pregnant women any of the following?	***n*** = **99 LAs**	***n*** = **30 NHS Trusts**
*Provide e-cigarettes, either directly or indirectly, e.g., by voucher*	11 (11%)	2 (7%)
*Incentives for success in quit attempts, e.g., money, shopping vouchers (please give details of incentives and value below)*	14 (14%)	5 (17%)
*None of the above*	67 (82%)	23 (77%)

## Data Availability

The data presented in this study are available on request from the corresponding author. The data are not publicly available as consent was not obtained to hold them in a public repository.

## Supplementary Materials

The following supporting information can be downloaded at: https://www.mdpi.com/article/10.3390/ijerph19031634/s1, Figure S1: LA Survey; Figure S2: NHS Trust Survey.
