# Supplementary material for: Smoking Cessation Support for Pregnant Women Provided by English Stop Smoking Services and National Health Service Trusts: A Survey
Source: Int J Environ Res Public Health. 2022 Jan 31;19(3):1634. doi: 10.3390/ijerph19031634 (PMC8835166; doi:10.3390/ijerph19031634)
Supplement: Supplementary file 1 [file ijerph-19-01634-s001.zip › Figure S1-LA Survey.pdf]

Questions on services for pregnant women, in partnership with the University of Nottingham

The following additional questions have been provided by the Smoking in Pregnancy Research Group at the University of Nottingham and will be used by the researchers to identify sites for a trial on supporting pregnant women's adherence to Nicotine Replacement Therapy (NRT) and quitting smoking. Further information on the trial is available here: https://bit.ly/2OKrE3J

The data collected below will be shared by ASH with Nottingham University. The data will not be published in the annual ASH local tobacco control report.

Unless stated otherwise all questions refer to the commissioning cycle April 2020 – March 2021.

If you have any queries relating to this survey please contact Ross Thomson on 0115 74 86677, email: ross.thomson1@nottingham.ac.uk

Part 2 Service set up

1. Does your local authority currently commission intensive stop smoking support for pregnant smokers?

- ◯ Yes
- ◯ No
- ◯ Don't know

2. In which of the following ways does your local authority currently provide support for pregnant smokers? (tick all that apply)

- ☐ Specialist stop smoking service
- ☐ Integrated lifestyle service
- ☐ Smoking cessation support in primary care
- ☐ Self-support (e.g. website, apps)
- ☐ Other (please describe)

Part 2 Staffing and practitioner consultations

3. Who provides smoking cessation support for the majority of pregnant women using your service? (tick all that apply)

- ☐ Stop smoking specialist(s) working only or mainly with pregnant women
- ☐ Stop smoking specialist(s) working with all smokers, including pregnant women
- ☐ Lifestyle/wellbeing counsellor(s)
- ☐ GPs or pharmacists
- ☐ Other (please describe)

4. What is the approximate number of full time equivalents (FTE) staff, indicated in the previous question, within your service who support pregnant women?

5. What smoking cessation training have advisors who see pregnant women undergone? (tick all that apply)

- ☐ ‘Very Brief Advice on Smoking’
- ☐ ‘Very Brief Advice on Smoking for Pregnant Women’
- ☐ ‘Stop Smoking Practitioner Training’
- ☐ ‘Pregnancy and Smoking Cessation’
- ☐ Face to face training delivered by NCSCT trained staff
- ☐ Don’t know
- ☐ Other (please describe)

6. How are initial stop smoking support appointments with pregnant women conducted? (tick all that apply)

- [ ] Individual face-to-face
- [ ] Group face-to-face
- [ ] Remote (e.g telephone, video call)
- [ ] Other (please describe)

7. How are follow-up stop smoking support appointments with pregnant women conducted? (tick all that apply)

- [ ] Individual face-to-face
- [ ] Group face-to-face
- [ ] Remote (e.g. telephone, video call)
- [ ] Other (please describe)

Part 2 NRT and smoking cessation aids

8. Does your service have a budget for or provide NRT to pregnant women?

- ◯ Yes
- ◯ No

Part 2 NRT and smoking cessation aid

9. How is NRT supplied to pregnant women? (tick all that apply)

- [ ] Direct provision of NRT from your service
- [ ] Voucher for NRT to be redeemed at a pharmacy
- [ ] GP prescription
- [ ] Other (please describe)

10. In total, how many weeks NRT does your service offer individual pregnant women? (give number of weeks)

11. What types of NRT does your service offer pregnant women? (tick all that apply)

- [ ] Patches
- [ ] Fast-acting NRT (e.g. mouth spray, gum, lozenge)
- [ ] Combination of patches and a fast acting NRT

For the next question, we define a brief smoking lapse as when someone who is otherwise abstinent in a quit attempt smokes a small amount of tobacco, even a single 'puff'.

For this the purposes of this survey, if brief smoking lapses continue each day for two weeks or more, we consider that the quitter has re-started smoking and she is no longer in a quit attempt.

12. How does your service expect practitioners to advise pregnant women who have brief smoking lapses on whether they should continue or stop using NRT?

- ( ) No particular expectation
- ( ) Continue using NRT
- ( ) Stop using NRT
- ( ) Other (please describe)

13. Does your service offer pregnant women any of the following? (tick all that apply)

- [ ] Provide e-cigarettes, either directly or indirectly e.g. by voucher
- [ ] Incentives for success in quit attempts e.g. money, shopping vouchers (please give details of incentives and value below)
- [ ] None of the above

Description of incentives

Part 2 Data collection

14. For all women that set a quit date, does your service routinely collect self-reported or CO-validated smoking status at any of the following times? (tick all that apply)

NB: only tick if your service attempts to get information from all women at a time point

- [ ] 4 weeks
- [ ] 12 weeks
- [ ] 6 months
- [ ] Other (please describe)

Part 2 Final items

15. When does the current tender for your stop smoking service expire?

Part 2 Future research

We have developed the 'Baby, Me & NRT' intervention which is designed to increase pregnant women's adherence to Nicotine Replacement Therapy when used to stop smoking.

The final part of this process is a randomised controlled trial (RCT) testing whether 'Baby, Me & NRT' helps pregnant women to quit.

This trial could run in your area; if it did, your SSS staff would benefit from free training and the pregnant women you see could benefit from extra support to help them to quit.

16. Would you like to hear more about this trial?

- ◯ Yes
- ◯ No

Thank you for completing the Local Authority survey of Stop Smoking Services for Pregnant Women.
Your input is much appreciated.
