# Supplementary material for: Smoking Cessation Support for Pregnant Women Provided by English Stop Smoking Services and National Health Service Trusts: A Survey
Source: Int J Environ Res Public Health. 2022 Jan 31;19(3):1634. doi: 10.3390/ijerph19031634 (PMC8835166; doi:10.3390/ijerph19031634)
Supplement: Supplementary file 1 [file ijerph-19-01634-s001.zip › Figure S2-NHS Trust Survey.pdf]

Maternity Survey Final

Page 1

Maternity Services Survey of Stop Smoking Support for Pregnant Women

The Smoking in Pregnancy research group at the University of Nottingham is working with ASH to find out more about how pregnant women who smoke are supported to stop and to build a comprehensive picture of where and how pregnant women are being supported. The results will also be used by the researchers to identify sites for a trial on supporting pregnant women's adherence to Nicotine Replacement Therapy (NRT) and quitting smoking. Further information on the trial is available here: https://bit.ly/2OKrE3J

The survey is comprised of around 28 brief questions.

Your progress through the survey is shown at the top of each page. If you are not able to complete this all in one sitting, you can click 'Finish later' at the bottom of the page. This will save your answers and you will then be able to close your browser and return to complete it later. This might be useful if, for example, you need to refer to other sources or people. If you *do* need to return to the survey at a later time you will need to use the link generated when you click 'Finish later'. Please note, we will not be able to see any of your answers until you click 'Finish' at the very end of the survey.

Unless stated otherwise all questions refer to the period between **April 2020 and March 2021**

All survey responses are collected over encrypted (SSL) connections. Your responses will remain confidential; your name will not be used and you will not be identifiable in any publications arising from the survey.

If you have any queries relating to this survey please contact Ross Thomson, email: ross.thomson1@nottingham.ac.uk

Service Set-up

*The following questions are about **intensive stop smoking support** (not just brief advice), which is **offered by the NHS to pregnant women**. Many NHS trusts will have started providing such support in response to the NHS Long Term Plan.*

***Please do not answer about similar support commissioned by local authorities**; a separate survey asking about this is being sent to local authorities.*

1. Does your NHS trust currently commission intensive stop smoking support for pregnant smokers? ✱ *Required*

- ○ Yes
- ○ No
- ○ Don’t know

Service Set-up

If you answered 'don’t know' to this question could you please forward the introductionary email to the person who may be able to answer this question and copy in the sender of that message (Efe.Mamuzo@ash.org.uk).

If you are unsure who can answer this question, please let us know at the same email address.

Thank you for your time.

You may click the FINISH button and leave the survey

Service Set-up

2. Are you responsible for overseeing the day-to-day delivery of intensive smoking cessation support to pregnant women? ✱ *Required*

- ◯ Yes
- ◯ No

Service set-up

As you answered no to this question could you please forward the introductionary email to the person who may be more able to complete this survey and copy in the sender of that message (Efe.Mamuzo@ash.org.uk).

If you are unsure who can answer this question, please let us know at the same email address.

Thank you for your time.

You may click the FINISH button and leave the survey

No Service Details

3. As you answered **no** to the previous question, could you please provide your name and email address for our records? *Please click NEXT to submit your answers and exit the survey*

Service set-up

4. Please give the name of the NHS Trust(s) in which you oversee the delivery of intensive smoking cessation support to pregnant women ✱ *Required*

5. Is any of this support joint-funded with a Local Authority/ies? ✱ *Required*

- [ ] Yes
- [ ] No
- [ ] Not sure/don't know

6. What is your role in this / these NHS Trusts? ✱ *Required*

7. Does your NHS Trust provide intensive stop smoking support for pregnant women in any of the following local settings? (*tick all that apply*) ✱ *Required*

- [ ] Hospital clinic
- [ ] Community clinic
- [ ] Not sure/don’t know
- [ ] Other

7.a. If you selected Other, please specify:

Staffing and practitioner consultations

8. Who provides intensive stop smoking support to pregnant smokers in your Trust? (*tick all that apply*) ✱ *Required*

- ☐ Stop smoking practitioners who are not otherwise clinically trained
- ☐ Specially-trained midwife(ves)
- ☐ Specially-trained health care assistants
- ☐ Other

8.a. If you selected Other, please specify:

9. What is the approximate number of full time equivalent (FTE) staff, indicated in question 6 above, within your service who support pregnant women?

What smoking cessation training have advisors who see pregnant women undergone? (*tick all that apply*)

10. NCSCT (National Centre for Smoking Cessation and Training) online modules?

- ☐ 'Very Brief Advice on Smoking'
- ☐ 'Very Brief Advice on Smoking for Pregnant Women'
- ☐ 'Stop Smoking Practitioner Training'
- ☐ 'Pregnancy and Smoking Cessation'

10.a. Other training?

- ☐ Face to face training delivered by NCSCT trained staff
- ☐ Other
- ☐ Don’t know

10.a.i. If you selected Other, please specify:

11. *How* are **initial** smoking cessation appointments with pregnant women conducted? (*tick all that apply*) ✱ *Required*

- ☐ Individual face to face
- ☐ Group face to face
- ☐ Remote (e.g. telephone, video call)
- ☐ Other

11.a. If you selected Other, please specify:

12. *How* are **follow-up** appointments conducted? (*tick all that apply*) ✱ *Required*

- ☐ Individual face-to-face
- ☐ Group face to face
- ☐ Remote (e.g. telephone, video call)
- ☐ Other

12.a. If you selected Other, please specify:

NRT and smoking cessation aids

13. Does your Trust have a budget for or provide NRT to pregnant women who want to stop smoking? ✱ *Required*

- ○ Yes
- ○ No

NRT and smoking cessation aids

14. How is NRT made available to pregnant women? (*tick all that apply*) ✱ *Required*

- ☐ Direct provision of NRT from practitioner
- ☐ Voucher for NRT to be supplied by pharmacy
- ☐ Hospital prescription (i.e. redeemed at hospital pharmacy)
- ☐ Other

14.a. If you selected Other, please specify:

15. In total, how many weeks of NRT does your Trust offer individual pregnant women (give number of weeks)? ✱ *Required*

16. What types of NRT does your Trust offer pregnant women? (*tick all that apply*) ✱ *Required*

- ○ Patches ONLY
- ○ Fast-acting NRT (e.g. mouth spray, gum, lozenge) ONLY
- ○ Combination of patches and a fast acting NRT together

NRT and smoking cessation aids

17. As your Trust does not provide NRT, are women recommended to obtain this from elsewhere? ✱ *Required*

- ○ Yes
- ○ No

NRT and smoking cessation aids

18. How are women recommended to obtain NRT? (tick all that apply) ✱ *Required*

- ☐ GP prescription
- ☐ Over the counter
- ☐ Local Authority Stop Smoking Service
- ☐ Other

18.a. If you selected Other, please specify:

NRT and smoking cessation aids

*For Q19, we define a **brief smoking lapse** as when someone who is otherwise abstinent in a quit attempt smokes a small amount of tobacco, even a single 'puff'.*

*For the purposes of this survey, if brief smoking lapses continue each day for **two weeks or more**, we consider that the quitter has re-started smoking and she is no longer in a quit attempt.*

19. How does your service expect practitioners to advise **pregnant women** who have **brief smoking lapses** on whether they should continue or stop using NRT? ✱ *Required*

- ◯ No particular expectation
- ◯ continue using NRT
- ◯ Stop using NRT
- ◯ Other

19.a. If you selected Other, please specify:

20. Does your service offer pregnant women any of the following? (tick all that apply) ✱ *Required*

- ◯ Provide e-cigarettes, either directly or indirectly (e.g. by voucher)
- ◯ Incentives for success in quit attempts e.g. money, shopping vouchers (please give details of incentives and value below)
- ◯ None of the above

20.a. Please give details of incentives and value

Data collection

21. Each year, approximately how many pregnant women set a quit date after receiving your Trust's stop smoking support? *N.B. If yours is a new service, how many women do you envisage setting a quit date in your first year* ✱ *Required*

22. For all women that set a quit date, does your Trust routinely collect **self-reported** smoking status at the following times? (*tick all that apply*) ✱ *Required*

- ☐ 4 weeks
- ☐ 12 weeks
- ☐ 6 months
- ☐ Other

22.a. If you selected Other, please specify:

23. For all women that set a quit date, does your Trust routinely collect **CO-validated** smoking status at the following times? (*tick all that apply*) ✱ *Required*

- ☐ 4 weeks
- ☐ 12 weeks
- ☐ 6 months
- ☐ Other

23.a. If you selected Other, please specify:

24. Is client data stored electronically? ✱ *Required*

- ◯ Yes
- ◯ No

25. Is it possible to identify pregnant women who have used your Trust’s stop smoking support within your database? ✱ *Required*

- ◯ Yes
- ◯ No

26. If you know it, please could you name the database software used? (e.g. Quit manager) or answer 'don't know' ✱ *Required*

Final items

27. When is the Trust set to recommission the intensive stop smoking support which is currently provided to pregnant women? ✱ *Required*

28. Are there any major changes to the way the Trust delivers stop smoking support to pregnant women which are likely to be in place within the next 12 months? **This does not include changes due to coronavirus.** ✱ *Required*

- Yes
- No planned changes

28.a. If you answered yes, could you briefly explain what those changes will be?

Future research

We have developed the ‘Baby, Me & NRT’ intervention which is designed to increase pregnant women’s adherence to Nicotine Replacement Therapy when used to stop smoking.

The final part of this process is a randomised controlled trial (RCT) testing whether ‘Baby, Me & NRT’ helps pregnant women to quit.

This trial could run in your area; if it did, your staff would benefit from free training and the pregnant women you see could benefit from extra support to help them to quit.

29. Would you like to hear more about this trial? ✱ *Required*

- ○ Yes
- ○ No

Your survey responses will be kept confidential. However, if you are happy to provide your contact details below, this will permit the research team to get in touch and tell you more about the trial.

30. Name

31. Email address

32. Telephone number

Submit your answers

Thank you for completing the Maternity Services Survey of Stop Smoking Support for Pregnant Women.

Your input is much appreciated

Please click 'Finish' to submit your responses

Final page

If you have any queries relating to this survey please contact:

Ross Thomson on 0115 74 86677

Email: ross.thomson1@nottingham.ac.uk
